# Internal Consistency and Structural Validity of the Norwegian Translation of the Ten-Item Personality Inventory

**DOI:** 10.3389/fpsyg.2021.723852

**Published:** 2021-08-11

**Authors:** Mikkel Magnus Thørrisen, Talieh Sadeghi, Jannecke Wiers-Jenssen

**Affiliations:** ^1^Department of Occupational Therapy, Prosthetics and Orthotics, Faculty of Health Sciences, OsloMet – Oslo Metropolitan University, Oslo, Norway; ^2^Department of Public Health, Faculty of Health Sciences, University of Stavanger, Stavanger, Norway; ^3^Nordic Institute for Studies in Innovation, Research and Education (NIFU), Oslo, Norway; ^4^Centre for the Study of Professions, OsloMet – Oslo Metropolitan University, Oslo, Norway

**Keywords:** big five, five-factor model, internal consistency, personality assessment, psychometric properties, structural validity, ten-item personality inventory

## Abstract

**Background:** The Ten-Item Personality Inventory (TIPI) is a validated brief instrument measuring the five-factor model (FFM) personality dimensions, developed for instances where more comprehensive FFM instruments are impractical to use. The TIPI has been translated into several languages, but psychometric properties of the Norwegian version (N-TIPI) have not been systematically explored.

**Objectives:** This study aimed to explore the psychometric properties of the N-TIPI, in terms of internal consistency and structural validity.

**Methods:** In a cross-sectional study, responses on the N-TIPI were collected from 5,009 Norwegian master graduates. Descriptive statistics for the subscales and correlations between subscales were calculated. Internal consistency was assessed with inter-item correlations, Cronbach’s α and Spearman-Brown coefficients. Structural validity was explored with principal component analysis, parallel analysis, and visual scree plot inspection. Results for the N-TIPI were compared with those previously reported for the original TIPI as well as the German, French, Spanish, and Portuguese versions.

**Results:** Compared with the original and non-English versions of TIPI, results for N-TIPI showed comparable subscale rank order of means, standard deviations, and pattern of correlations between subscales, as well as inter-item correlations and Cronbach’s α. The 10 N-TIPI items were adequately reduced to five components, theoretically corresponding with the FFM personality domains.

**Conclusion:** The N-TIPI demonstrated acceptable internal consistency and satisfactory structural validity. Although further research is warranted, the instrument stands out as feasible when it is essential to minimize participants’ response burden in studies that aim to explore personality as one among several concepts or utilize personality traits as covariates.

## Introduction

The five-factor model (FFM) of personality (McCrae and Costa, 2008), often referred to as the Big Five, represents the predominant model for capturing and understanding individual differences in personality (John et al., 2008). The FFM assumes that personality is organized in five broad domains: extraversion (E), agreeableness (A), conscientiousness (C), neuroticism (N; also oppositely named emotional stability, ES), and openness to experience (O; John et al., 2008). The FFM taxonomy has demonstrated cross-cultural replicability (McCrae et al., 1998), and a large body of evidence has suggested that personality traits predict a variety of life outcomes, such as health, longevity, marital success, and educational as well as occupational attainment (Ozer and Benet-Martinez, 2006; Roberts et al., 2007). In research, measurement of personality is expedient for a variety of purposes across fields and topic areas.

Several instruments for measuring the FFM domains have been developed and validated, including the 240-item NEO Personality Inventory-Revised (NEO-PI-R; Costa and McCrae, 1992), the 60-item NEO Five-Factor Inventory (NEO-FFI; Costa and McCrae, 1992), and the 44-item Big-Five Inventory (BFI; John and Srivastava, 1999). The most comprehensive instrument, the NEO-PI-R, takes approximately 45 min to complete (Gosling et al., 2003), which is often considered too lengthy in research that does not solely focus on personality exploration. In many instances, even the NEO-FFI and BFI may be considered too comprehensive, taking approximately 15 and 5 min to complete, respectively (John and Srivastava, 1999). Researchers may want to study personality as one among several concepts, or simply control for participants’ personality characteristics (Storme et al., 2016), which can be difficult by means of relatively lengthy instruments. In practice, “circumstances are often not ideal and researchers may be faced with a stark choice of using an extremely brief instrument or using no instrument at all” (Gosling et al., 2003, p. 505).

Gosling et al. (2003) developed the Ten-Item Personality Inventory (TIPI) as a brief and time-efficient measure of the FFM personality domains. The TIPI comprises a total of 10 items. More specifically, the instrument consists of five two-item subscales corresponding to the FFM domains, scored on a seven-point Likert scale. The original psychometric evaluation of the TIPI (Gosling et al., 2003) concluded that this brief instrument constitutes a reasonable proxy for more comprehensive FFM instruments, e.g., by demonstrating acceptable convergent and discriminant validity, test–retest reliability, and patterns of external correlations. Also, the TIPI has been used to measure personality states in experience sampling studies (e.g., Sosnowska et al., 2020). The TIPI has been utilized comprehensively, which is reflected in that the instrument’s validation article (Gosling et al., 2003) has been cited more than 7,500 times in the literature.

The TIPI has later been translated into a variety of languages, and several non-English versions of the instrument have demonstrated acceptable psychometric properties, including translations into Bangla (Islam, 2019), Catalan (Renau et al., 2013), French (Storme et al., 2016), Georgian (Martskvishvili et al., 2020), German (Muck et al., 2007), Indonesian (Akhtar, 2018), Japanese (Iwasa and Yoshida, 2018), Persian (Azkhosh et al., 2020), Portuguese (Nunes et al., 2018), and Spanish (Romero et al., 2012; Renau et al., 2013).

A Norwegian version of the TIPI (N-TIPI) was developed by Cristina Aicher and is available online (Gosling, n.d.). According to Bergvik and Wynn (2012, p. 392), the N-TIPI “was developed by using standard translation back-translation procedures by bilingual native English and native Norwegian researchers.” The N-TIPI has been utilized in research, for instance in studies of study and work addiction (Atroszko et al., 2016), the use of digital technology among hospitalized patients (Bergvik and Wynn, 2012), pandemic behavior (Götz et al., 2021), and anxiety and depression among odontology students (Risstad et al., 2017). However, the internal consistency and structural validity of the N-TIPI have not been systematically explored.

### Study Aim

The aim of this study was to explore the psychometric properties of the Norwegian version of the TIPI (N-TIPI), in terms of internal consistency and structural validity. Psychometric properties of the N-TIPI were compared with the original version (Gosling et al., 2003; Ehrhart et al., 2009) as well as with four non-English translations: German (Muck et al., 2007), French (Storme et al., 2016), Portuguese (Nunes et al., 2018), and Spanish (Renau et al., 2013). These non-English versions were selected based on them being properly validated. It was *a priori* assumed that it was more suitable to compare the Norwegian translation with other European languages than for instance with Bangla, Indonesian, or Persian translations.

## Materials and Methods

### Design and Setting

This study was designed as a cross-sectional psychometric assessment of the N-TIPI, conducted in a sample of 5,009 Norwegian master graduates.

### Data Collection and Sample

Data were obtained from the Norwegian graduate survey, a digital survey conducted among Norwegian graduates with a master degree or equivalent in 2019, 3 years upon graduation, by the Nordic Institute for Studies in Innovation, Research and Education (NIFU; Skjelbred, 2019). The eligible sample included all individuals who had graduated from Norwegian higher education institutions in 2016 (*n* = 12,578) as well as Norwegian citizens who had graduated from higher education institutions abroad in 2015 and 2016 (*n* = 5,018). Hence, a total of 17,596 individuals were invited, and 6,188 (35.2%) provided informed consent and agreed to participate. According to Skjelbred et al. (2019), individuals who agreed to participate were substantially representative of the population. However, 1,179 graduates failed to respond on all relevant study items and were excluded, leaving a final study sample of 5,009 individuals.

Females constituted the majority of the sample (62.4%), individuals aged 28–30 years represented the most common age group (49.3%), and social sciences/law (22.1%) and natural/technical sciences (21.7%) were the most prevalent fields of study among the participants. Characteristics of the study sample are presented in Table 1.

**Table 1:** Characteristics of the study sample (*n* = 5,009).

Variable	N	%
Gender		
Female	3127	62.4
Male	1881	37.6
Age		
≤27 years	507	10.1
28–30 years	2468	49.3
31–35 years	1224	24.4
≥36 years	810	16.2
Field of study		
Humanities/arts	509	10.2
Teacher training/pedagogy	491	9.8
Social sciences/law	1108	22.1
Business and administration	802	16.0
Science and technology	1088	21.7
Health/social/sports (not medicine)	576	11.5
Medicine	372	7.4
Primary industries/transport/communications	58	1.2
Other	5	0.1

### Instrument: The N-TIPI

The N-TIPI was administered to participants by means of a digital survey. The 10 items are designed to measure the FFM personality domains (E, A, C, ES, and O), each domain with two items (Gosling et al., 2003). Participants were asked to indicate, on a seven-point Likert scale (1 = disagree strongly; 2 = disagree moderately; 3 = disagree a little; 4 = neither agree nor disagree; 5 = agree a little; 6 = agree moderately; and 7 = agree strongly), the extent to which they agreed that a set of 10 descriptive statements applied to them. For instance, “extraverted, enthusiastic” (Norwegian: “utadvendt, entusiastisk”) and “reserved, quiet” (Norwegian: “reservert, stille”) represented the indicators of the E domain. An overview of items, item wordings, and response categories for the N-TIPI and the original TIPI is presented in Table 2.

**Table 2:** Overview of items, item wordings, and response categories in the original version (TIPI) and the Norwegian translation (N-TIPI).

Item	Domain	Wording (original version)^a^	Wording (Norwegian translation)^b^
TIPI-1	E	Extraverted, enthusiastic	Utadvendt, entusiastisk
TIPI-2 (R)	A	Critical, quarrelsome	Kritisk, kverulerende
TIPI-3	C	Dependable, self-disciplined	Pålitelig, selvdisiplinert
TIPI-4 (R)	ES	Anxious, easily upset	Engstelig, lett opprørt
TIPI-5	O	Open to new experiences, complex	Åpen for nye erfaringer, kompleks
TIPI-6 (R)	E	Reserved, quiet	Reservert, stille
TIPI-7	A	Sympathetic, warm	Sympatisk, varm
TIPI-8 (R)	C	Disorganized, careless	Uorganisert, uvøren
TIPI-9	ES	Calm, emotionally stable	Rolig, emosjonelt stabil
TIPI-10 (R)	O	Conventional, uncreative	Konvensjonell, lite kreativ
Response categories:		1 = disagree strongly; 2 = disagree moderately; 3 = disagree a little; 4 = neither agree nor disagree; 5 = agree a little; 6 = agree moderately; 7 = agree strongly	1 = meget uenig; 2 = uenig; 3 = litt uenig; 4 = hverken enig eller uenig; 5 = litt enig; 6 = enig; 7 = veldig enig

*R, reverse-scored item; E, extraversion; A, agreeableness; C, conscientiousness; ES, emotional stability; O, openness*.

a *Original TIPI (Gosling et al., 2003)*.

b *Norwegian translation, developed by Cristina Aicher (Gosling, n.d.)*.

The five subscales were constructed with the following procedure (Gosling et al., 2003): First, reverse-scored items (N-TIPI-2, N-TIPI-4, N-TIPI-6, N-TIPI-8, and N-TIPI-10) were recoded, ensuring that higher scores indicated higher domain values. Second, a mean score was calculated for each subscale.

### Analysis

The 10 N-TIPI items were analyzed descriptively by calculating means (*M*) and standard deviations (*SD*), separately for the 10 items as well as for the five subscales. Descriptive statistics for the N-TIPI subscales were compared with results reported for the original TIPI (Gosling et al., 2003), the German TIPI-G (Muck et al., 2007), the French TIPI-F (Storme et al., 2016), the Spanish TIPI-SPA (Renau et al., 2013), and the Portuguese TIPI-P (Nunes et al., 2018). The N-TIPI pattern of subscale correlations was calculated and compared to those reported for the original TIPI (Ehrhart et al., 2009), and for the TIPI-G and TIPI-F.

Internal consistency of the N-TIPI was assessed by calculating inter-item correlations (Pearson *r*), Cronbach’s α, and Spearman-Brown coefficients (S-B) for each of the five two-item subscales. N-TIPI estimates of internal consistency were compared with those reported for the original TIPI, TIPI-G, TIPI-F, TIPI-SPA, and TIPI-P. Although S-B coefficients are generally considered more appropriate than Pearson *r* and Cronbach’s α for estimating internal consistency of two-item scales (Eisinga et al., 2013), the two latter statistics were also applied in this study to enable comparisons with the other TIPI versions.

The structural validity of the N-TIPI was assessed with exploratory principal component analysis (PCA) using orthogonal (varimax) rotation. Parallel analysis (Horn, 1965) and visual scree plot inspection (Cattell, 1966) were performed to aid in determining the adequate number of components to extract. It was *a priori* defined that a fully satisfactory component structure had to meet the following six criteria:

1. The 10 items had to be suitable for PCA, as indicated by a statistically significant Bartlett’s test of sphericity (*p* < 0.05; Bartlett, 1954) and a Kaiser-Meyer-Olkin measure of sampling adequacy (KMO) reaching ≥0.50 (Kaiser, 1974; Hutcheson and Sofroniou, 1999);
2. the component structure had to demonstrate five components with Eigenvalues (*λ*) ≥1.00 (Kaiser, 1960), and the extraction of five components had to be supported by both parallel analysis and visual scree plot inspection;
3. the five-component structure had to explain ≥50% of the variance in the data, and each of the five components had to explain ≥10% of the variance (Merenda, 1997);
4. the 10 items had to load pairwise on the five components, theoretically in accordance with the FFM personality domains;
5. each item had to load substantially (≥0.40) on its corresponding FFM component (Ford et al., 1986; Hair et al., 1998), without any cross-loadings, i.e., without loadings of ≥0.32 on two or more components (Costello and Osborne, 2005); and
6. each item communality (*h*^2^) had to reach ≥0.20 (Child, 2006).

An exploratory analysis was chosen in order to investigate whether it was possible to generate a FFM with a bottom-up (data driven) approach that satisfied the abovementioned six criteria, rather than to simply examine properties of a pre-defined five-factor solution (confirmatory analysis). Orthogonal rotation was chosen based on an *a priori* assumption that components would not be correlated at ≥0.32 (Tabachnick and Fidell, 2013).

All analyses were performed with IBM SPSS version 27, with the exception of parallel analysis that was conducted with an engine developed by Patil et al. (2017). The analytical procedures were based on the COSMIN guidelines for evaluating internal consistency and structural validity of measurement instruments (Terwee et al., 2012).

### Ethics

Participants were assured confidentiality and informed that participation was voluntary. Written informed consent was obtained from all participants (see Skjelbred et al., 2019 for more details).

## Results

### Descriptive Statistics

Means and standard deviations for the N-TIPI items are presented in Table 3.

**Table 3:** Descriptive statistics for the N-TIPI items.

Item	Domain	*M* (*SD*)	Item	Domain	*M* (*SD*)
TIPI-1	E	5.22 (1.51)	TIPI-6 (R)	E	4.54 (1.72)
TIPI-2 (R)	A	3.96 (1.61)	TIPI-7	A	5.58 (1.15)
TIPI-3	C	6.02 (1.01)	TIPI-8 (R)	C	5.53 (1.46)
TIPI-4 (R)	ES	4.88 (1.61)	TIPI-9	ES	5.42 (1.28)
TIPI-5	O	5.82 (1.00)	TIPI-10 (R)	O	4.84 (1.47)

*M, mean; SD, standard deviation; R, reverse-scored item; E, extraversion; A, agreeableness; C, conscientiousness; ES, emotional stability; O, openness*.

Of the N-TIPI subscales, the highest mean scores were found for C (*M* = 5.78; *SD* = 1.07), followed by O (*M* = 5.33; *SD* = 0.99), ES (*M* = 5.15; *SD* = 1.24), E (*M* = 4.88; *SD* = 1.45), and A (*M* = 4.77; *SD* = 1.09). Descriptive statistics for the N-TIPI subscales – compared with the original TIPI, TIPI-G, TIPI-F, TIPI-SPA, and TIPI-P – are presented in Table 4.

**Table 4:** Descriptive statistics (means and standard deviations) and rank order of means for the TIPI subscales, N-TIPI compared with the original TIPI and four other versions.

Instrument	TIPI subscales				
	C	O	ES	E	A
	*M* (*SD*)	*M* (*SD*)	*M* (*SD*)	*M* (*SD*)	*M* (*SD*)
**N-TIPI (Norwegian)**	**5.78 (1.07)**	**5.33 (0.99)**	**5.15 (1.24)**	**4.88 (1.45)**	**4.77 (1.09)**
**subscale rank order**	**1**	**2**	**3**	**4**	**5**
TIPI (original)^a^	5.40 (1.32)	5.38 (1.07)	4.83 (1.42)	4.44 (1.45)	5.23 (1.11)
*subscale rank order*	*1*	*2*	*4*	*5*	*3*
TIPI-G (German)^b^	5.85 (0.93)	5.49 (0.97)	5.10 (1.20)	4.87 (1.21)	5.20 (0.95)
*subscale rank order*	*1*	*2*	*4*	*5*	*3*
TIPI-F (French)^c^	5.72 (1.15)	5.34 (1.13)	4.72 (1.38)	4.54 (1.42)	5.37 (1.05)
*subscale rank order*	*1*	*3*	*4*	*5*	*2*
TIPI-SPA (Spanish)^d^	5.13 (1.25)	5.33 (1.12)	4.35 (1.30)	5.10 (1.35)	4.38 (0.80)
*subscale rank order*	*2*	*1*	*5*	*3*	*4*
TIPI-P (Portuguese)^e^	5.64 (1.21)	5.30 (1.22)	3.79 (1.30)	4.52 (1.56)	6.09 (0.84)
*subscale rank order*	*2*	*3*	*5*	*4*	*1*

*C, conscientiousness; O, openness; ES, emotional stability; E, extraversion; A, agreeableness; M, mean; SD, standard deviation*.

a *Gosling et al. (2003)*.

b *Muck et al. (2007)*.

c *Storme et al. (2016)*.

d *Renau et al. (2013)*.

e *Nunes et al. (2018)*.

As shown in Table 4, the N-TIPI subscales demonstrated a comparable rank order of means and standard deviations as the original TIPI, TIPI-G, and TIPI-SPA, i.e., with higher mean scores for C and O than for ES, E, and A. TIPI-F and TIPI-P deviated from this pattern by having higher mean scores for A and C than for O, ES, and E.

### Correlations Between Subscales

All five N-TIPI subscales were significantly correlated, yet none of the subscales were correlated ≥0.32. In line with the original TIPI, all subscale associations were positive and the correlation between E and O was the strongest (*r* = 0.28, *p* < 0.001 for the N-TIPI). Correlations between N-TIPI subscales – compared with the original TIPI, TIPI-G, and TIPI-F – are presented in Table 5.

**Table 5:** Correlations between the TIPI-subscales, N-TIPI compared with the original TIPI and two other versions.

Instrument	TIPI subscales				
		A	C	ES	O
N-TIPI (Norwegian)	E	**0.08**	**0.08**	**0.13**	**0.28**
	A		**0.19**	**0.09**	**0.08**
	C			**0.18**	**0.05**
	ES				**0.12**
TIPI (original)^1^	E	0.05	0.04	0.15	0.35
	A		0.12	0.27	0.21
	C			0.18	0.21
	ES				0.22
TIPI-G (German)^2^	E	−0.03	0.09	0.33	0.42
	A		0.25	0.10	0.16
	C			0.39	0.21
	ES				0.20
TIPI-F (French)^3^	E	0.01	−0.07	0.03	0.26
	A		0.16	0.28	0.15
	C			0.19	0.04
	ES				0.15

*E = extraversion; A = agreeableness; C = conscientiousness; ES = emotional stability*.

1 *Ehrhart et al. (2009)*,

2 *Muck et al. (2007), and*

3 *Storme et al. (2016)*.

As shown in Table 5, the pattern of correlations between the N-TIPI subscales was quite comparable to those reported for the original TIPI, TIPI-G, and TIPI-F.

### Internal Consistency

Internal consistency was highest for the E subscale (*r* = 0.61, *p* < 0.001; *α* = 0.75; S-B = 0.76), followed by ES (*r* = 0.47, *p* < 0.001; *α* = 0.62; S-B = 0.64), C (*r* = 0.47, *p* < 0.001; *α* = 0.61; S-B = 0.62), O (*r* = 0.27, *p* < 0.001; *α* = 0.41; S-B = 0.43), and A (*r* = 0.22, *p* < 0.001; *α* = 0.35; S-B = 0.36). Internal consistency for the N-TIPI subscales – compared with the original TIPI, TIPI-G, TIPI-F, TIPI-SPA, and TIPI-P – are presented in Table 6.

**Table 6:** Internal consistency (correlations, alpha coefficients, and Spearman-Brown coefficients) for the TIPI subscales, N-TIPI compared with the original TIPI and four other versions.

Instrument	TIPI subscales									
	E		A		C		ES		O	
	*r*	α	*r*	α	*r*	α	*r*	α	*r*	α
**N-TIPI (Norwegian)**	**0.61**	**0.75**	**0.22**	**0.35**	**0.47**	**0.61**	**0.47**	**0.62**	**0.27**	**0.41**
*Spearman-Brown*	*0.76*		*0.36*		*0.64*		*0.64*		*0.43*	
TIPI (original)^1^	0.59	0.68	0.36	0.40	0.42	0.50	0.61	0.74	0.28	0.45
TIPI-G (German)^2^	–	0.57	–	0.42	–	0.66	–	0.67	–	0.54
TIPI-F (French)^3^	0.52	0.69	0.13	0.22	0.40	0.57	0.44	0.61	0.23	0.39
TIPI-SPA (Spanish)^4^	0.46	0.71	0.16	0.08	0.42	0.48	0.29	0.51	0.38	0.51
TIPI-P (Portuguese)^5^	–	0.72	–	0.39	–	0.45	–	0.43	–	0.60

*E, extraversion; A, agreeableness; C, conscientiousness; ES, emotional stability; r, correlation coefficient; α, alpha coefficient (Cronbach’s α)*.

1 *Gosling et al. (2003)*,

2 *Muck et al. (2007)*,

3 *Storme et al. (2016)*,

4 *Renau et al. (2013), and*

5 *Nunes et al. (2018)*.

As evident in Table 6, N-TIPI subscale inter-item correlations (range: *r* = 0.22 to 0.61) and estimates of Cronbach’s α (range: *α* = 0.35 to 0.75) were quite comparable to those reported for the original TIPI (*r* = 0.28 to 0.61; *α* = 0.40 to 0.74) and TIPI-G (*α* = 0.42 to 0.67). The internal consistency of N-TIPI was somewhat higher than results reported for the TIPI-F (*r* = 0.13 to 0.52; *α* = 0.22 to 0.69), TIPI-SPA (*r* = 0.16 to 0.46; *α* = 0.08 to 0.71), and TIPI-P (*α* = 0.39 to 0.72).

### Structural Validity

The N-TIPI demonstrated satisfactory structural validity. The 10 items were deemed suitable for PCA (Table 7), as indicated by a statistically significant Bartlett’s test of sphericity (*p* < 0.001) and a KMO of 0.56. The PCA identified five components with *λ* exceeding 1.0 (C_1_ = 2.24; C_2_ = 1.63; C_3_ = 1.29; C_4_ = 1.06; C_5_ = 1.03).

**Table 7:** Component structure for the 10 N-TIPI items.

Items	Components					Communality
	C_1_	C_2_	C_3_	C_4_	C_5_	
	E	C	ES	A	O	
TIPI-6 “Reserved, quiet”	**0.91**	0.00	0.09	0.04	0.00	0.84
TIPI-1 “Extraverted, enthusiastic”	**0.83**	0.08	−0.02	0.26	0.06	0.77
TIPI-3 “Dependable, self-disciplined”	0.00	**0.86**	0.08	0.12	0.05	0.76
TIPI-8 “Disorganized, careless”	0.07	**0.83**	0.08	−0.11	0.07	0.71
TIPI-4 “Anxious, easily upset”	0.24	0.02	**0.86**	0.03	−0.06	0.81
TIPI-9 “Calm, emotionally stable”	−0.15	0.16	**0.81**	0.10	0.13	0.73
TIPI-7 “Sympathetic, warm”	0.06	0.09	0.11	**0.83**	−0.02	0.68
TIPI-2 “Critical, quarrelsome”	0.17	−0.08	0.02	**0.66**	0.04	0.77
TIPI-5 “Open to new experiences, complex”	−0.04	−0.02	0.18	−0.18	**0.84**	0.71
TIPI-10 “Conventional, uncreative”	0.14	0.22	−0.15	0.33	**0.69**	0.48
	**C_1_**	**C_2_**	**C_3_**	**C_4_**	**C_5_**	**All**
	E	C	N	A	O	
Eigenvalue λ	2.24	1.63	1.29	1.06	1.03	−
% explained variance	22.36	16.25	12.89	10.64	10.25	72.40

*Component structure generated with exploratory principal component analysis (PCA) with orthogonal (varimax) rotation; Kaiser-Meyer Olkin measure of sampling adequacy (KMO) = 0.56; and Bartlett’s test of sphericity: χ^2^ (45) = 8050.76, p < 0.001*.

A parallel analysis (Table 8) indicated that only components with an *λ* of ≥1.01 should be retained. Hence, parallel analysis supported the extraction of five components (for the sixth component, the randomly generated *λ* exceeded the corresponding *λ* in the data: *λ*6_random_ = 0.99; *λ*6_data_ = 0.86).

**Table 8:** Parallel analysis for the N-TIPI component structure.

	*λ* dataset	λ randomly generated
Component 1	**2.24**	1.07
Component 2	**1.63**	1.05
Component 3	**1.29**	1.03
Component 4	**1.06**	1.02
Component 5	**1.03**	1.01
Component 6	0.86	0.99
Component 7	0.64	0.98
Component 8	0.49	0.97
Component 9	0.42	0.95
Component 10	0.34	0.93

*N = 5,009; N variables = 10; N random correlation matrices = 100; and Percentiles of eigenvalues = 95*.

**Figure 1:**
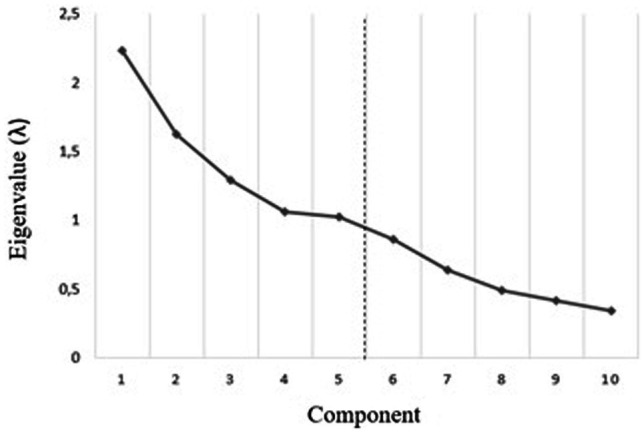
Scree plot.

Although somewhat unclear, the scree plot (Figure 1) indicated an inflection between the fifth and sixth components, supporting the extraction of five components.

As shown in Table 7, the five-component structure explained a total of 72.4% of the variance in the data, and each of the five components explained more than 10% of the variance (C_1_ = 22.36%; C_2_ = 16.25%; C_3_ = 12.89%; C_4_ = 10.64%; C_5_ = 10.25%). The 10 N-TIPI items loaded pairwise on the five components, theoretically in accordance with the FFM personality domains. Each item loaded substantially (≥0.40) on its corresponding FFM component without any cross-loadings. Each item communality (*h*^2^) reached ≥0.20 (range: *h*^2^ = 0.48 to 0.84).

In sum, the results showed that the N-TIPI met the six *a priori* defined criteria for a fully satisfactory component structure. First, preliminary analyses yielded suitability for PCA. Second, a five-component structure was supported by PCA, parallel analysis, and visual scree plot inspection. Third, the five-component structure explained more than 50% of the variance in the data and each of the components explained at least 10% of the variance. Fourth, the items loaded pairwise on the five components in accordance with the FFM personality domains and each item loaded substantially on its corresponding FFM component without any cross-loadings. Finally, each item communality reached ≥0.20.

## Discussion

The aim of this study was to explore the psychometric properties of the Norwegian version of the TIPI (N-TIPI). Results showed that the N-TIPI demonstrated acceptable psychometric properties in terms of internal consistency and structural validity.

Compared to the original TIPI (Gosling et al., 2003), the German TIPI-G (Muck et al., 2007), and the Spanish TIPI-SPA (Renau et al., 2013), the N-TIPI demonstrated a comparable rank order of subscale means and standard deviations. TIPI-F (Storme et al., 2016) and TIPI-P (Nunes et al., 2018) displayed a somewhat different pattern, which can be attributed to measurement issues, or may reflect actual cultural differences between countries. The N-TIPI also showed a comparable pattern of correlations between subscales with those reported for the original TIPI (Ehrhart et al., 2009), TIPI-G, and TIPI-F (Storme et al., 2016). Moreover, the N-TIPI demonstrated satisfactory structural validity: The 10 N-TIPI items could be adequately reduced to five components, theoretically corresponding with the FFM personality domains (E, A, C, N/ES, and O).

Previous research on the TIPI has indicated that this brief instrument constitutes a reasonable proxy for more comprehensive FFM instruments (Gosling et al., 2003), such as the 240-item NEO-PI-R and 60-item NEO-FFI (Costa and McCrae, 1992) as well as the 44-item BFI (John and Srivastava, 1999). Response burden is an important consideration when designing questionnaires. Research has indicated that participation rates have been declining over time (Galea and Tracy, 2007; Boyle et al., 2021) and a systematic review and meta-analysis of the relationship between response rate and questionnaire length found that longer questionnaires were associated with lower response rates (Rolstad et al., 2011). Response rate is seen as an indicator of study quality, and low response rates constitute a concern for external validity, i.e., for the “generalizability of findings *to* and *across* target populations” (Pedhazur and Schmelkin, 1991, p. 229). The N-TIPI may thus be a serviceable alternative to more comprehensive FFM instruments when it is essential to minimize participants’ response burden in studies that aim to explore personality as one among several concepts or utilize personality as a covariate.

Although the N-TIPI generated estimates of internal consistency comparable to those reported for the original TIPI, TIPI-G, TIPI-F, TIPI-SPA, and TIPI-P, low internal consistency (e.g., low Cronbach’s α coefficients for the subscales) has been emphasized as a limitation applying to most versions of this brief instrument (Storme et al., 2016). It should be noted, however, that it is far from straightforward to adequately assess internal consistency for two-item scales. Scholars disagree on which measures are most appropriate in such instances. While Cronbach’s α is the most frequently applied statistic, some argue that Pearson correlation is more appropriate, yet others advocate the utilization of Spearman-Brown coefficients (Eisinga et al., 2013). Eisinga et al. (2013, p. 641) conclude that the latter is most serviceable to two-item scales: “[T]he Spearman-Brown coefficient is never lower than coefficient alpha and almost always higher. It is also on average less biased, especially if the correlation between the items is relatively strong.” In this study of the N-TIPI, we assessed subscale internal consistency in terms of Pearson correlations, Cronbach’s α, and Spearman-Brown coefficients. Unfortunately, Spearman-Brown coefficients are not reported for the other versions of TIPI. Hence, we were only able to compare inter-item correlations and α coefficients.

The current exploration of measurement properties of the N-TIPI assumed a reflective approach based on an assumption that items were correlated and constituted effects of common latent factors (Markus and Borsboom, 2013). Hence, it was deemed appropriate to assess internal consistency and component structure. One may argue that the TIPI (in line with other FFM instruments) is based on a reflective model, which is evident in that the validation of the original instrument focused on reflective statistical procedures, such as inter-item correlations and Cronbach’s α (Gosling et al., 2003). However, rather than emphasizing internal consistency, the original TIPI was designed with an aim of maximizing content validity in order to capture the breadth of the FFM domains (Gosling et al., 2003). Therefore, one may not expect the TIPI to reach commonly accepted thresholds of internal consistency. As noted by Chiorri et al. (2014, p. 110), the developers could have tackled this problem by using “items with a very high correlation (e.g., *r* > 0.70), which, given their unavoidable redundancy, would have undermined content coverage.” According to DeVellis (2003), satisfactory scale internal consistency is indicated by Cronbach’s α reaching ≥0.70. Given that the TIPI consists of two-item subscales, and since the instrument was not designed to maximize internal consistency, it was in this study more pivotal to compare the internal consistency of N-TIPI with other validated versions of TIPI rather than with conventional thresholds for acceptable scale reliability. Alternatively, measurement properties of the N-TIPI could have been explored with a formative approach, i.e., assuming that items did not necessarily correlate and that they constituted samples of particular behaviors rather than effects of common latent factors (Markus and Borsboom, 2013). For instance, Myszkowski et al. (2019) demonstrated that a formative approach had merits in comparison with a traditional reflective approach for short scales with a broad content, such as the TIPI.

### Methodological Considerations and Implications for Future Research

This is the first study to systematically explore psychometric properties of the Norwegian version of the TIPI (N-TIPI). We were able to demonstrate acceptable internal consistency and satisfactory structural validity of the N-TIPI. The study sample was large (*n* = 5,009) and substantially representative of the eligible sample (Skjelbred et al., 2019). However, certain limitations should be kept in mind when interpreting results from this study. First, the sample consisted solely of individuals who had completed a master’s degree or equivalent, and participants were thus far higher educated than the general Norwegian population. Second, due to the study’s cross-sectional design and certain data limitations (N-TIPI was the only FFM instrument in the survey), we were not able to assess test–retest reliability of the N-TIPI or convergent validity with other validated FFM instruments.

This study represents an important step on the path to a fully validated Norwegian version of the TIPI. Future research on the N-TIPI could benefit from utilizing general population samples, exploring test–retest reliability and convergent validity with other validated Norwegian FFM instruments, i.e., the NEO-PI-R, NEO-FFI, and BFI (Martinsen et al., 2003, 2005; Engvik and Føllesdal, 2005). A large and growing number of TIPI versions have been and are being developed, and this brief FFM instrument is widely utilized in research. Future research on the TIPI could benefit from secondary research efforts (e.g., systematic or scoping reviews) focusing on providing an overview of translated and validated versions and their psychometric properties.

### Conclusion

This was the first study to systematically explore psychometric properties of the N-TIPI. The N-TIPI demonstrated acceptable internal consistency and satisfactory structural validity. Although further research on the N-TIPI is warranted, the instrument stands out as feasible when it is essential to minimize participants’ response burden in studies that aim to explore personality as one among several concepts or utilize personality traits as covariates.

## Data Availability Statement

Publicly available datasets were analyzed in this study. The data analyzed in this study was obtained from the Norwegian Centre of Research Data (NSD; https://doi.org/10.18712/NSD-NSD2941-V1). Requests to access this dataset should be directed to nsd@nsd.no.

## Ethics Statement

The studies involving human participants were reviewed and approved by Norwegian Centre for Research Data. The patients/participants provided their written informed consent to participate in this study.

## Author Contributions

This study was designed by MT, TS, and JW-J. MT analyzed the data and drafted the manuscript. TS and JW-J provided scientific input to the different drafts and provided data interpretation. All authors made critical revisions and provided intellectual content to the manuscript, approved the final version to be published, and agreed to be accountable for all aspects of this work.

## Conflict of Interest

The authors declare that the research was conducted in the absence of any commercial or financial relationships that could be construed as a potential conflict of interest.

## Publisher’s Note

All claims expressed in this article are solely those of the authors and do not necessarily represent those of their affiliated organizations, or those of the publisher, the editors and the reviewers. Any product that may be evaluated in this article, or claim that may be made by its manufacturer, is not guaranteed or endorsed by the publisher.
